# Supplementary material for: A Comprehensive Analysis of Plasma Cytokines and Metabolites Shows an Association between Galectin-9 and Changes in Peripheral Lymphocyte Subset Percentages Following Coix Seed Consumption
Source: Nutrients. 2022 Apr 19;14(9):1696. doi: 10.3390/nu14091696 (PMC9102546; doi:10.3390/nu14091696)
Supplement: Supplementary file 1 [file nutrients-14-01696-s001.zip › Table S1.pdf]

Table S1 List of kits used to analyze the cytokine concentration

Kit	Cytokine
Human Th Cytokine Panel	IL-5, IL-13, IL-2, IL-6, IL-9, IL-10, IFN-γ, TNF-α, IL-17A, IL-17F, IL-4, IL-22
Human Cytokine Panel 2	TSLP, IL-1α, IL-1β, GM-CSF, IFN-α2, IL-23, IL-12p40, IL-12p70, IL-15, IL-18, IL-11, IL-27, IL-33
Human CD8-NK Panel	sFas, sFasL, Granzyme A, Granzyme B, Perforin, Granulysin
Human Checkpoint Panel 1	sCD25, 4-1BB, sCD27, B7.2, TGF-β1 (Free Active), CTLA-4, PD-L1, PD-L2, PD-1, Tim-3, LAG-3, Galectin-9
Human Proinflammatory Panel 1	IL-8, IP-10, Eotaxin, TARC, MCP-1, RANTES, MIP-1α, MIG, ENA-78, MIP-3α, GROα, I-TAC, MIP-1β
