# Supplementary material for: A Comprehensive Analysis of Plasma Cytokines and Metabolites Shows an Association between Galectin-9 and Changes in Peripheral Lymphocyte Subset Percentages Following Coix Seed Consumption
Source: Nutrients. 2022 Apr 19;14(9):1696. doi: 10.3390/nu14091696 (PMC9102546; doi:10.3390/nu14091696)
Supplement: Supplementary file 1 [file nutrients-14-01696-s001.zip › Table S2.pdf]

Table S2 Plasma cytokine concetrations

| | | Coix seed | | | | | | Control | | | | | |
|---|---|---|---|---|---|---|---|---|---|---|---|---|---|
| | | Pre | | Post | | | | Pre | | Post | | | |
| Cyto-ID | Name | Mean | SD | Mean | SD | p-value | | Mean | SD | Mean | SD | p-value | |
| 1 | IL-5 (pg/mL) | 1.583 | 1.579 | 1.597 | 1.845 | 0.7874 | | 1.518 | 0.608 | 1.746 | 1.114 | 0.4443 | |
| 2 | IL-13 (pg/mL) | 7.43 | 8.188 | 7.449 | 7.737 | 1.0000 | | 10.051 | 4.734 | 10.189 | 5.268 | 0.8989 | |
| 3 | IL-2 (pg/mL) | 3.386 | 5.545 | 3.281 | 5.124 | 1.0000 | | 1.573 | 0.509 | 1.752 | 0.703 | 0.3597 | |
| 4 | IL-6 (pg/mL) | 2.841 | 3.296 | 2.166 | 2.214 | 0.5286 | | 2.565 | 0.8 | 6.016 | 10.461 | 0.9453 | |
| 5 | IL-9 (pg/mL) | 1.989 | 1.841 | 1.691 | 1.696 | 0.0801 | | 1.83 | 0.721 | 1.819 | 0.853 | 0.9378 | |
| 6 | IL-10 (pg/mL) | 0.54 | 0.36 | 0.456 | 0.318 | 0.1415 | | 0.666 | 0.424 | 0.601 | 0.266 | 0.5469 | |
| 7 | IFN-γ (pg/mL) | 4.59 | 8.251 | 4.455 | 8.021 | 0.4753 | | 2.407 | 2.098 | 2.938 | 1.766 | 0.2945 | |
| 8 | TNF-α (pg/mL) | 6.792 | 3.568 | 2.704 | 2.673 | 0.0020 | ** | 9.527 | 5.652 | 2.69 | 2.366 | 0.0234 | * |
| 9 | IL-17A (pg/mL) | 0.276 | 0.303 | 0.298 | 0.313 | 0.2719 | | 0.202 | 0.136 | 0.375 | 0.46 | 0.5294 | |
| 10 | IL-17F (pg/mL) | 1.607 | 2.193 | 1.58 | 2.391 | 0.5281 | | 4.041 | 6.136 | 4.351 | 7.5 | 0.7422 | |
| 11 | IL-4 (pg/mL) | 3.311 | 5.269 | 3.223 | 5.012 | 0.7893 | | 2.726 | 2.09 | 3.31 | 2.424 | 0.2012 | |
| 12 | IL-22 (pg/mL) | 2.018 | 4.001 | 1.135 | 1.362 | 0.3096 | | 0.596 | 0.351 | 0.728 | 0.579 | 0.4925 | |
| 13 | TSLP (pg/mL) | 5.104 | 6.86 | 4.6 | 6.239 | 0.2084 | | 3.947 | 5.693 | 4.85 | 8.226 | 0.5896 | |
| 14 | IL-1α (pg/mL) | 37.788 | 42.039 | 33.276 | 40.084 | 0.2049 | | 54.122 | 45.05 | 54.111 | 53.596 | 0.7422 | |
| 15 | IL-1β (pg/mL) | 9.471 | 12.865 | 8.272 | 12.006 | 0.0801 | | 11.307 | 14.421 | 12.887 | 21.842 | 0.4002 | |
| 16 | GM-CSF (pg/mL) | 3.604 | 6.894 | 2.896 | 6.247 | 0.0756 | | 7.385 | 12.616 | 9.078 | 18.443 | 1.0000 | |
| 17 | IFN-α2 (pg/mL) | 8.74 | 12.596 | 7.809 | 11.102 | 0.4922 | | 13.844 | 19.107 | 15.3 | 25.755 | 0.6406 | |
| 18 | IL-23 (pg/mL) | 17.78 | 24.769 | 15.607 | 22.976 | 0.1073 | | 7.358 | 7.568 | 6.761 | 7.019 | 0.7874 | |
| 19 | IL-12p40 (pg/mL) | 368.414 | 435.124 | 339.762 | 413.921 | 0.5566 | | 582.291 | 797.486 | 645.626 | 1027.384 | 1.0000 | |
| 20 | IL-12p70 (pg/mL) | 3.086 | 7.502 | 2.851 | 6.265 | 1.0000 | | 1.95 | 3.004 | 3.336 | 5.511 | 0.1057 | |
| 21 | IL-15 (pg/mL) | 124.323 | 144.205 | 118.526 | 135.775 | 0.5896 | | 121.219 | 126.648 | 143.787 | 146.675 | 0.0935 | |
| 22 | IL-18 (pg/mL) | 106.747 | 32.743 | 102.621 | 39.53 | 0.5165 | | 94.796 | 32.279 | 94.53 | 37.005 | 0.9642 | |
| 23 | IL-11 (pg/mL) | 58.423 | 64.521 | 55.387 | 59.317 | 0.9442 | | 59.224 | 58.995 | 72.305 | 74.688 | 0.0346 | * |
| 24 | IL-27 (pg/mL) | 37.651 | 27.292 | 34.647 | 31.864 | 0.0840 | | 48.172 | 69.755 | 66.133 | 122.266 | 0.7422 | |
| 25 | IL-33 (pg/mL) | 115.561 | 124.633 | 102.284 | 116.863 | 0.0840 | | 189.766 | 150.957 | 200.745 | 214.557 | 0.3828 | |
| 26 | sFas (pg/mL) | 464.096 | 173.161 | 423.123 | 213.74 | 0.2671 | | 534.895 | 327.705 | 473.541 | 318.508 | 0.2297 | |
| 27 | sFasL (pg/mL) | 14.202 | 16.614 | 13.961 | 15.812 | 0.8336 | | 11.919 | 11.897 | 9.478 | 8.781 | 0.2767 | |
| 28 | Granzyme A (pg/mL) | 33.918 | 43.911 | 36.228 | 48.773 | 0.5566 | | 34.718 | 19.313 | 37.095 | 18.455 | 0.5469 | |
| 29 | Granzyme B (pg/mL) | 442.523 | 598.019 | 470.658 | 608.885 | 0.1551 | | 361.68 | 182.342 | 377.337 | 242.579 | 0.6853 | |
| 30 | Perforin (pg/mL) | 1160.17 | 254.821 | 1089.799 | 262.711 | 0.1681 | | 1553.938 | 568.468 | 1306.663 | 310.501 | 0.1137 | |
| 31 | Granulysin (pg/mL) | 1362.035 | 584.072 | 1236.16 | 566.357 | 0.0177 | * | 1251.207 | 353.024 | 1195.626 | 278.356 | 0.3960 | |
| 32 | sCD25 (IL-2Ra) | 403.288 | 192.1 | 344.777 | 106.083 | 0.0371 | * | 381.585 | 148.286 | 390.705 | 169.936 | 0.7309 | |
| 33 | 4-1BB (pg/mL) | 125.825 | 148.912 | 120.596 | 138.492 | 0.4316 | | 285.12 | 335.715 | 288.921 | 389.214 | 0.4609 | |
| 34 | sCD27 (pg/mL) | 16901.79 | 5130.348 | 15185.19 | 5108.296 | 0.0087 | ** | 16075.84 | 4472.859 | 14340.6 | 2940.917 | 0.0645 | |
| 35 | B7.2 (pg/mL) | 119.586 | 31.025 | 112.681 | 29.667 | 0.0497 | * | 104.954 | 52.349 | 104.704 | 64.895 | 0.4609 | |
| 36 | TGF-β1(Free Active) | 123.96 | 35.797 | 140.593 | 52.455 | 0.1756 | | 141.769 | 46.861 | 156.799 | 57.856 | 0.3572 | |
| 37 | CTLA-4 (pg/mL) | 3.236 | 6.265 | 3.311 | 5.724 | 0.1309 | | 6.115 | 11.253 | 7.916 | 16.871 | 0.9453 | |
| 38 | PD-L1 (pg/mL) | 26.335 | 50.125 | 24.927 | 45.844 | 0.4316 | | 37.905 | 52.609 | 42.989 | 73.065 | 0.6406 | |
| 39 | PD-L2 (pg/mL) | 3409.385 | 897.79 | 3100.019 | 864.241 | 0.0024 | ** | 3452.927 | 910.039 | 3168.057 | 727.724 | 0.0422 | * |
| 40 | PD-1 (pg/mL) | 21.876 | 25.595 | 20.586 | 24.29 | 0.0840 | | 46.585 | 65.462 | 52.174 | 90.362 | 0.2500 | |
| 41 | Tim-3 (pg/mL) | 1135.758 | 343.528 | 1069.307 | 513.495 | 0.6250 | | 1306.001 | 496.269 | 1231.905 | 602.651 | 0.3334 | |
| 42 | LAG-3 (pg/mL) | 974.04 | 887.598 | 858.48 | 843.609 | 0.0059 | ** | 1523.65 | 1782.27 | 1567.275 | 2148.765 | 0.7422 | |
| 43 | Galectin-9 (pg/mL) | 20522.77 | 4604.374 | 18982.87 | 4809.372 | 0.0390 | * | 21600.66 | 5630.121 | 22487.98 | 7507.335 | 0.5870 | |
| 44 | IL-8 (pg/mL) | 45.01 | 42.048 | 23.249 | 12.161 | 0.0098 | ** | 51.822 | 41.884 | 37.705 | 49.843 | 0.0391 | * |
| 45 | IP-10 (pg/mL) | 42.53 | 15.942 | 38.008 | 15.648 | 0.0730 | | 33.032 | 11.838 | 34.54 | 11.069 | 0.3637 | |
| 46 | Eotaxin (pg/mL) | 26.856 | 5.633 | 25.317 | 5.982 | 0.0063 | ** | 27.711 | 9.639 | 25.637 | 9.06 | 0.1025 | |
| 47 | TARC (pg/mL) | 160.8 | 92.081 | 131.36 | 50.44 | 0.0840 | | 100.95 | 32.591 | 94.994 | 37.408 | 0.3976 | |
| 48 | MCP-1 (pg/mL) | 155.256 | 47.011 | 146.923 | 41.755 | 0.1211 | | 167.1 | 21.484 | 143.936 | 19.07 | 0.0664 | |
| 49 | RANTES (pg/mL) | 1589.879 | 534.273 | 1393.553 | 465.832 | 0.0488 | * | 2475.471 | 1999.592 | 1363.781 | 612.868 | 0.0656 | |
| 50 | MIP-1α (pg/mL) | 46.864 | 39.994 | 31.732 | 25.262 | 0.0422 | * | 50.209 | 42.623 | 45.774 | 46.875 | 0.6726 | |
| 51 | MIG (pg/mL) | 9.197 | 8.165 | 8.51 | 10.547 | 0.4316 | | 8.268 | 6.223 | 7.161 | 3.235 | 0.5443 | |
| 52 | ENA-78 (pg/mL) | 194.579 | 83.054 | 188.503 | 78.765 | 0.2754 | | 190.644 | 113.32 | 188.225 | 118.503 | 0.7422 | |
| 53 | MIP-3α (pg/mL) | 7.702 | 8.796 | 4.38 | 4.043 | 0.1925 | | 5.156 | 2.784 | 5.856 | 4.01 | 0.2620 | |
| 54 | GROα (pg/mL) | 43.306 | 11.6 | 41.594 | 11.222 | 0.0268 | * | 46.075 | 12.12 | 44.224 | 8.499 | 0.4609 | |
| 55 | I-TAC (pg/mL) | 19.15 | 16.572 | 16.576 | 11.955 | 0.3223 | | 16.787 | 16.096 | 19.418 | 23.161 | 0.3828 | |
| 56 | MIP-1β (pg/mL) | 15.962 | 6.686 | 11.808 | 3.477 | 0.0208 | * | 18.446 | 8.274 | 14.329 | 8.97 | 0.0341 | * |

*, $p < 0.05$; **, $p < 0.01$
