# Supplementary material for: A Comprehensive Analysis of Plasma Cytokines and Metabolites Shows an Association between Galectin-9 and Changes in Peripheral Lymphocyte Subset Percentages Following Coix Seed Consumption
Source: Nutrients. 2022 Apr 19;14(9):1696. doi: 10.3390/nu14091696 (PMC9102546; doi:10.3390/nu14091696)
Supplement: Supplementary file 1 [file nutrients-14-01696-s001.zip › Table S3.pdf]

Table S3 Plasma metabolite concetrations

		Coix seed						Control					
		Pre		Post				Pre		Post			
Chem_ID	Name	Mean	SD	Mean	SD	p-value		Mean	SD	Mean	SD	p-value	
1	Ornithine	86.152	12.299	87.322	10.957	0.6271		76.913	6.197	72.315	13.837	0.2830	
2	Choline	21.995	2.881	21.728	2.26	0.9219		23.253	3.333	22.704	5.073	0.7858	
3	Lysine	246.675	22.396	229.857	31.276	0.1086		212.361	35.421	209.681	27.344	0.6406	
4	Arginine	74.117	9.865	74.046	12.735	0.9750		88.868	17.377	85.385	13.182	0.3896	
5	Histidine	77.232	9.903	77.952	12.741	0.7515		71.096	6.898	67.037	12.847	0.2993	
6	Creatinine	66.355	8.19	59.674	5.94	0.0017	**	64.205	8.077	60.045	10.131	0.0414	*
7	beta-Alanine	5.602	1.264	6.979	2.572	0.1754		5.614	1.442	4.949	2.046	0.6406	
8	GABA	9.284	6.928	11.157	7.594	0.0239	*	10.46	5.78	12.469	6.57	0.1443	
9	Glycine	267.182	37.898	279.169	50.271	0.2325		264.442	24.31	274.993	23.758	0.1704	
10	Creatine	24.767	7.11	25.94	12.676	1.0000		20.057	9.508	18.308	3.983	0.3828	
11	Alanine	386.799	48.539	409.935	75.933	0.1342		359.164	44.575	360.086	67.01	0.9538	
12	Sarcosine	1.543	0.564	1.065	1.262	0.2863		1.61	0.699	0.788	0.597	0.0547	
13	Serine	134.712	18.43	133.084	24.08	0.7253		126.493	5.349	130.861	13.625	0.4176	
14	Valine	230.077	41.23	220.656	26.711	0.3247		216.842	33.295	215.664	26.985	0.9278	
15	Isoleucine	61.295	12.907	56.348	10.958	0.3429		57.214	6.913	56.422	7.428	0.9453	
16	Leucine	135.868	21.281	132.39	15.906	0.3960		128.007	13.926	124.824	12.649	0.6961	
17	Asparagine	51.43	7.123	52.845	5.869	0.4620		47.476	6.318	47.652	7.432	0.9431	
18	Threonine	138.446	24.025	143.914	32.126	0.5242		116.032	16.371	113.544	22.65	0.7488	
19	Anthranilic acid	0.323	0.164	0.148	0	0.0084	**	0.35	0.358	0.148	0	0.1558	
20	Methionine	23.598	3.522	20.997	2.503	0.1202		21.096	2.117	18.283	3.095	0.0452	*
21	Glutamine	453.049	38.75	474.219	49.404	0.1341		468.709	47.288	467.022	66.434	0.9326	
22	Proline	155.442	33.371	165.175	33.056	0.3138		157.559	34.979	161.95	45.936	0.6672	
23	Dimethylglycine	3.829	1.257	3.78	0.749	0.8673		4.012	0.974	4.594	1.395	0.2370	
24	Glutamic acid	177.621	32.691	190.262	41.07	0.2539		191.162	31.296	207.068	41.475	0.3585	
25	Tryptophan	56.025	9.952	60.571	9.835	0.2312		51.753	8.416	55.982	8.017	0.2019	
26	Hypoxanthine	13.112	2.303	13.35	2.663	0.7141		12.074	1.942	14.323	1.423	0.0051	**
27	Citrulline	26.376	6.821	29.838	5.495	0.0241	*	28.127	4.522	28.002	5.617	0.9536	
28	Phenylalanine	60.702	8.367	86.05	49.047	0.0645		60.891	6.941	72.341	12.814	0.0645	
29	Betaine	45.25	10.86	46.373	9.615	0.7776		55.544	17.104	54.639	15.624	0.8495	
30	Cysteine	3.932	1.159	2.625	1.049	0.0144	*	3.63	0.697	2.19	1.345	0.0582	
31	Tyrosine	56.729	6.997	60.116	7.922	0.1537		54.341	5.363	53.403	4.307	0.7168	
32	Aspartic acid	20.444	2.555	20.29	3.383	0.8706		21.474	3.753	23.227	3.554	0.3041	
33	Hydroxyproline	18.201	7.753	18.047	6.951	1.0000		17.312	6.21	15.632	4.797	0.5417	
34	Uracil	3.246	0.959	2.569	1.257	0.1953		3.727	1.109	3.307	0.762	0.3828	
35	Inosine	1.889	1.54	0.887	1.384	0.1055		0.559	0.363	2.157	1.464	0.0179	*
36	Uridine	9.943	1.168	9.391	1.816	0.4112		10.925	2.216	10.207	1.904	0.6406	
37	Gluconic acid	2.437	0.254	2.463	0.335	0.7727		3.152	1.614	3.463	1.627	0.1484	
38	3-Hydroxybutyric acid	68.277	70.284	59.229	45.023	0.4316		80.078	109.002	93.743	132.494	0.6406	
39	2-Hydroxybutyric acid	45.52	14.984	41.422	17.635	0.3490		33.818	17.319	45.885	28.604	0.0781	
40	2-Oxoisovaleric acid	11.726	2.527	13.746	3.15	0.0893		10.548	2.196	12.719	3.213	0.1550	
41	Lactic acid	2357.051	365.685	2578.58	356.212	0.1857		2481.512	595.633	2562.396	484.297	0.5357	
42	D-Ribulose 5-phosphate	0.477	0.316	0.333	0.453	0.1551		0.271	0.216	0.569	0.451	0.1240	
43	Glycolic acid	40.741	17.426	53.421	16.734	0.0490	*	34.722	9.372	53.225	15.079	0.0391	*
44	Pyruvic acid	27.451	9.63	35.462	25.727	0.4316		28.478	13.701	25.916	11.951	0.6695	
45	3-phosphoglyceric acid	0.189	0.192	0.102	0.065	0.2936		0.12	0.099	0.092	0.06	0.9453	
46	Succinic acid	6.518	0.488	6.892	1.237	0.3935		6.631	0.601	7.217	1.929	0.3884	
47	Malic acid	3.227	0.679	4.204	0.76	< 0.0001	****	3.183	0.403	4.617	1.337	0.0369	*
48	2-Oxoglutaric acid	0.223	0.115	0.535	0.695	0.2807		0.198	0.073	0.549	0.329	0.0591	
49	Fumaric acid	0.76	0.445	0.531	0.391	0.4069		0.808	0.461	0.693	0.546	0.7364	
50	Citric acid	56.834	13.97	64.985	10.435	0.0800		53.587	7.881	69.819	18.359	0.0078	**
51	cis-Aconitic acid	1.041	0.344	1.411	0.339	0.0023	**	1.018	0.386	1.415	0.462	0.1386	
52	Isocitric acid	1.27	0.425	1.611	0.393	0.0251	*	1.232	0.476	1.602	0.547	0.2261	

Concentratons: nmole/ml

*, p < 0.05; **, p < 0.01, ***, p < 0.001, ****, p < 0.0001
